# Phagocytosis converts infiltrated monocytes to microglia-like phenotype in experimental brain ischemia

**DOI:** 10.1186/s12974-022-02552-5

**Published:** 2022-07-18

**Authors:** Hyunwoo Ju, Keun Woo Park, Il-doo Kim, John W. Cave, Sunghee Cho

**Affiliations:** 1 Burke Neurological Institute, 785 Mamaroneck Ave, White Plains, NY 10605 USA; 2 InVitro Cell Research, Englewood, NJ USA; 3 Feil Brain Mind Research Institute, Weill Cornell Medicine, New York, NY USA

**Keywords:** Ischemic stroke, Phagocytes, Monocyte-derived macrophages, Microglia, Phagocytosis, Phenotype

## Abstract

**Background:**

Monocyte-derived macrophages (MDMs) and microglia elicit neural inflammation and clear debris for subsequent tissue repair and remodeling. The role of infiltrating MDMs in the injured brain, however, has been controversial due to overlapping antigen expression with microglia. In this study, we define the origin and function of MDMs in cerebral ischemia.

**Methods:**

Using adoptive transfer of GFP^+^ splenocytes into adult asplenic mice subjected to transient middle cerebral artery occlusion, we compared the role of CD11b^**+**^/CD45^+^/NK1.1^**−**^/Ly6G^**−**^ MDMs and microglia in the ischemic brain. The phagocytic activities of MDMs and microglia were measured by the uptake of fluorescent beads both in vivo with mice infused with GFP^+^ splenocytes and ex vivo with cultures of isolated brain immune cells.

**Results:**

Stroke induced an infiltration of MDMs [GFP+] into the ipsilateral hemisphere at acute (3 days) and sub-acute phases (7 days) of post-stroke. At 7 days, the infiltrating MDMs contained both CD45^High^ and CD45^Low^ subsets. The CD45^High^ MDMs in the injured hemisphere exhibited a significantly higher proliferation capacity (Ki-67 expression levels) as well as higher expression levels of CD11c when compared to CD45^Low^ MDMs. The CD45^High^ and CD45^Low^ MDM subsets in the injured hemisphere were approximately equal populations, indicating that CD45^High^ MDMs infiltrating the ischemic brain changes their phenotype to CD45^Low^ microglia-like phenotype. Studies with fluorescent beads reveal high levels of MDM phagocytic activity in the post-stroke brain, but this phagocytic activity was exclusive to post-ischemic brain tissue and was not detected in circulating monocytes. By contrast, CD45^Low^ microglia-like cells had low levels of phagocytic activity when compared to CD45^High^ cells. Both in vivo and ex vivo studies also show that the phagocytic activity in CD45^High^ MDMs is associated with an increase in the CD45^Low^/CD45^High^ ratio, indicating that phagocytosis promotes MDM phenotype conversion.

**Conclusions:**

This study demonstrates that MDMs are the predominant phagocytes in the post-ischemic brain, with the CD45^High^ subset having the highest phagocytic activity levels. Upon phagocytosis, CD45^High^ MDMs in the post-ischemic brain adopt a CD45^Low^ phenotype that is microglia-like. Together, these studies reveal key roles for MDMs and their phagocytic function in tissue repair and remodeling following cerebral ischemia.

**Supplementary Information:**

The online version contains supplementary material available at 10.1186/s12974-022-02552-5.

## Background

Stroke triggers a profound immune activation and a massive infiltration of peripheral immune cells, including monocytes, that leads to neural inflammation and brain injury [1–3]. Mouse and human monocytes are a heterogeneous population that includes pro-inflammatory (CCR2^**+/**^Ly-6C^High^) and reparative anti-inflammatory monocytes (CCR2^**−**^/Ly-6C^Low^) [4]. Stroke severity is closely associated with CCR2^+^ expression levels and the number of the monocytes in the post-ischemic brain [2, 3, 5]. Despite the negative association of the pro-inflammatory monocytes in aggravating brain damage, the absence of early monocyte recruitment in the injured CNS impairs regenerative processes and delays stroke recovery [6, 7]. This paradox suggests that monocyte-derived macrophages (MDMs) also have a beneficial function in resolving inflammation and promoting tissue repair, and that MDMs have a context-dependent role in stroke pathophysiology [1, 8–14].

The dichotomy of MDM function is reflected by their phenotypic heterogeneity. Whether classically or alternatively activated, MDMs can change their behavior and phenotype depending on ischemic milieu [15, 16]. In the post-ischemic brain, infiltrating CCR2^+^ monocytes differentiate into tissue macrophages with alternative M2 phenotype features [9]. Fate-mapping studies tracing monocytes based on CCR2 and/or CX3CR1 expression showed that monocytes in the injured brain can transition into microglia-like cells, revealing phenotype plasticity and functional convergence between MDMs and resident microglia [7, 17–19]. In acute experimental autoimmune encephalomyelitis, MDMs also acquire microglia-specific markers during chronic inflammation and disease-associated microglia adopt an inflammatory phenotype [15–17, 20]. These bidirectional changes in phenotype mediated by the tissue environment make it difficult to distinguish MDMs from resident microglia based on expression markers, which has obscured the origin and specific function of MDMs in the injured brain.

The clearance of cellular debris is a critical function of MDMs for tissue resolution and tissue remodeling. Phagocytic activity affects cellular metabolism [16, 21, 22] and macrophage function can be influenced by hypoxia or nutrient alterations [23–27]. In this current study, we sought to distinguish MDMs from microglia in the ischemic brain in order to define their function. We show that infiltrating MDMs in the post-ischemic brain convert to a microglia-like phenotype following phagocytic activity. We also show that MDMs, and not microglia, are the major phagocytic cell type in the injured brain the during acute/sub-acute phases. Together, the findings reveal an expanded role for peripheral immunity in CNS injury and repair processes.

## Material and methods

### Animals

Animals were maintained at a controlled temperature, humidity, and on a 12-h light/dark cycle. Each cage contained a maximum of 5 mice and was supplied with ventilation and irradiated bedding (1/8-in. Bed O’s Cobs, The Anderson, Maumee, OH). Sterilized food (PicoLab Rodent diet 5053, LabDiet, St. Louis, MO) and water were freely accessible in the cages. Male and female mice aged 3–4 months, C57BL/6 or GFP transgenic mice (C57BL/6-Tg (UBC-GFP), Jackson Laboratories, Bar Harbor, ME) were used.

### Transient middle cerebral artery occlusion (MCAO)

Procedures for 30-min transient MCAO and post-stroke care have been previously described [28]. Briefly, mice were anesthetized with an isoflurane/oxygen/nitrogen mixture. A 6-0 Teflon-coated black monofilament surgical suture (Doccol, Redland, CA) was inserted into the exposed external carotid artery and advanced into the internal carotid artery until it was wedged into the Circle of Willis, where it obstructs the origin of MCA. The filament was left in place for 30 min and then withdrawn. Cerebral blood flow (CBF) was measured prior to, during, and after stroke, and was monitored by Laser-Doppler flowmetry (Periflux System 5010; Perimed, Järfälla, Sweden). Only animals that had both > 80% reduction of pre-ischemic baseline CBF during MCAO and CBF > 80% of baseline after 10 min of reperfusion were included in the study. Body temperature was maintained at 37 ± 0.5 °C during, and for 30 min after, the MCA procedure via a rectal probe connected to a thermocouple-regulated heating water coil in the surgical board. Mice were placed in a recovery cage and their body temperatures were maintained at 37 ± 0.5 °C until they regained consciousness and resumed activity, after which they were returned to their home cages. Warm saline was administered subcutaneously to prevent dehydration during the acute phase. Softened food and hydrogel (Clear H_2_O) were given during the first week of recovery. Mice typically started to regain body weight around 3–5 days post-stroke and continued to recover.

### Splenectomy and adoptive transfer of splenocytes

Splenectomies and adoptive transfers of splenocytes in mice were performed as previously described [28]. Briefly, mice were anesthetized with isoflurane and a ~1-cm incision was made on the left side of the abdominal cavity under the rib cage. The spleen was removed by cutting the mesentery and connective tissue, and the splenic vessels were cauterized. Meloxicam (5 mg/kg, P.O.) was administered as an analgesic prior to surgery, in addition to buprenorphine (0.5 mg/kg, S.C., every 12 h for the first 48 h after surgery) and bupivacaine (0.1 ml of 0.25–0.5%, S.C., on incision site, before incision). For trafficking studies, splenocytes were harvested from GFP (green fluorescent protein) transgenic mice (C57BL/6-Tg(UBC-GFP)30Scha/J, Jackson laboratory) or C57BL/6 splenocytes labeled with green fluorescence by PKH67 (Green Fluorescent Cell Linker Kit; Sigma). The spleen was excised and minced using scissors, then pipetted in ice-cold Hanks' balanced salt solution (Life Technologies) without Ca^2+^ and Mg^2+^. The mixture passed through a 70 µm strainer and then centrifuged at 3000 rpm for 10 min at 4 °C. Erythrocytes were removed with Red Blood Cell Lysis buffer (Sigma). Isolated splenocytes (1–2 × 10^7^) were transferred into asplenic MCAO mice via the retro-orbital venous sinus and killed 24 h after for analyses.

### Isolation of brain immune cells

Mice were anesthetized with isoflurane and pentobarbital before being perfused with ice-cold phosphate-buffered saline (PBS) containing heparin. Brains were removed and the hemispheres were separated before being placed into ice-cold Hanks’ balanced salt solution without Ca^2+^ and Mg^2+^ (HBSS, Life Technologies, Grand Island, NY). Tissue was enzymatically and mechanically dissociated using a MACS Neural Tissue Dissociation Kit with Papain (Miltenyi Biotec, Bergisch Gladbach, Germany) and then treated with a myelin debris removal solution (Miltenyi Biotec). Isolated brain immune cells were used for either flow cytometer analyses or cultured for ex vivo phagocytic activity assays.

### Flow cytometry analysis

From single cell preparations, the total number of GFP^+^ cells from each hemisphere was determined by multiplying dilution factors to GFP^+^ event reads. For cell staining, primary antibodies used were: phycoerythrin CD11b (PE-Vio770 anti-mouse CD11b REA, 1:50); CD45 (Vio-blue anti-mouse CD45 REA, 1:50); Ki-67 (PE anti-mouse Ki67 REA, 1:50); CD11c (PE anti-mouse CD11c REA, 1:50); and a mixture of lineage markers (Lin) conjugated with allophycocyanin (APC) (APC-Lin) against T cells (APC anti-mouse CD90.2 REA), B cells (APC anti-mouse CD45R/B220 REA), natural killer cells (APC anti-mouse NK-1.1 REA, and APC anti-mouse CD49b REA), and granulocytes (APC anti-mouse Ly-6G REA) [29]. Following incubation at room temperature for 20 min in dark the cells were washed with PBS, and then analyzed with a MACS Quant VYB flow cytometer (Miltenyi Biotec, San Diego, CA). Antibodies used for flow cytometry were purchased from Miltenyi Biotec. Antibody specificity was determined by analyzing cell only, as well as single and double antibody controls for validation.

### Assessment of phagocytosis

Using a previously published protocol [1], immune cells (1 × 10^5^ cells/ well) from either brains or splenocytes were plated on 24-well plates (2 × 10^5^ cells/well) and incubated at 37 °C with 5% CO_2_ for 1 h. After washing away non-adherent cells, 1 μm (diameter) red fluorescent microsphere beads (beads^580/605^; F-13083 from ThermoFisher) were added to cell suspensions and incubated at 37 °C with 5% CO_2_ for 4 h. Non-phagocytosed beads were removed by multiple PBS washes and cells were resuspended in PBS. Phagocytic activity was determined by measuring the number of beads^580/605+^ cells, using cells incubated at 4 °C with beads as negative controls. For in vivo studies, a mixture of GFP^+^ splenocytes (1–2 × 10^7^ cells in 100 ul PBS) and beads^580/605^ (4 × 10^7^ beads in 100 ul PBS) was retro-orbitally injected into asplenic MCAO mice 1 day before killing. MDMs [GFP^+^] and microglia [GFP-] in the blood and brain were analyzed 24 h after infusion for incorporation of beads^580/605^.

### Immunohistochemistry

Mice were transcardially perfused with 4% paraformaldehyde in 0.1 mol/L phosphate buffer. Brains were collected and post-fixed overnight, placed into a 30% sucrose solution, and cryosectioned at a 30 µm thickness. Sections were washed in PBS, incubated with 1% bovine serum albumin and 5% normal goat serum for 1 h at room temperature, and incubated with anti-rabbit Iba-1 (1:1000, Wako, Richmond, VA, 019–19741) and anti-chicken GFP (1:1,000, Aves Lab, Davis, CA, AB16901) overnight at 4 °C. This was followed by incubation with either Alexa Fluor 488 goat anti-chicken IgG (1:2000, Life Technologies, A11039) or Alexa Fluor 594 goat anti-rabbit IgG (1:2000, Life Technologies, A11012) secondary antibodies for 1 h. After washing with PBS, the sections were mounted using Fluoroshield reagent (Sigma-Aldrich, F6057). Confocal image stacks were taken with an inverted A1R-HD25 confocal microscope (Nikon Instruments Inc., Melville, NY) using a 40x (NA 1.3) oil objective in 0.3 µm z-steps and at 0.43 µm/pixel. Displayed images represent maximum projections of the obtained z-stack for each imaged site and examined under a laser scanning confocal microscope (Carl Zeiss, Thornwood, NY, USA).

### Statistics

Comparisons between CD45 subsets were evaluated using Student’s *t* test. Multiple comparisons between groups were made using ANOVA followed by a post hoc Bonferroni comparison. Two-way ANOVA were performed for in vivo studies measuring the (1) effect of stroke and (2) effect of times as well as for ex vivo studies measuring the (1) effect of stroke and (2) effect of phagocytosis. Statistical analyses were performed using Prism software (GraphPad Software Inc., La Jolla, CA), and differences were considered significant if *p* < 0.05.

## Results

### MDMs enter the acute post-ischemic stroke brain

**Fig. 1:**
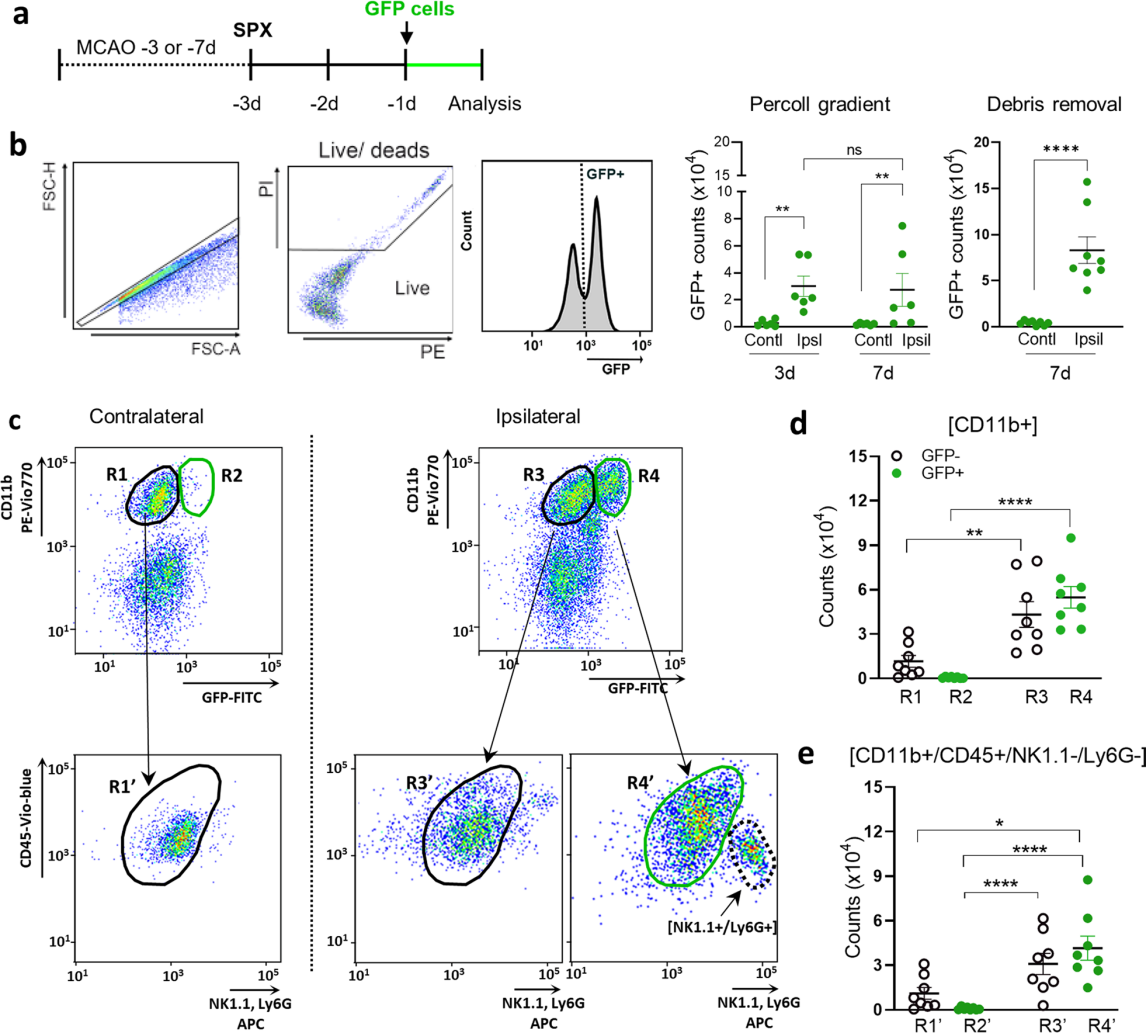
Stroke-induced monocyte trafficking to the brain. **a** Experimental timeline. Middle cerebral arterial occlusions (MCAO) were performed either 3 days (d) or 7d prior to killing. Spleens were removed (SPX) 3d prior to killing the mice. GFP^+^ splenocytes (1–2 × 10^7^) were infused retro-orbitally into asplenic mice at 1d prior to killing (or 2d post-splenectomy) so that GFP^+^ splenocytes circulated either during post-stroke days 2 and 3 for 3-day survival experiments or during post-stroke days 6 and 7 for 7-day survival experiments. **b** Flow cytometry and counts for GFP^+^ cells in the brain that were isolated by Percoll gradient at either 3 days (*n* = 6–7) and 7 days (*n* = 6), as well as for GFP^+^ cells isolated with debris removal method at 7 days (*n* = 6–7). Live cells were gated as negative for propidium iodine (PI) staining. **c** Gating strategy to identify mononuclear phagocytes at 7 days. R1–R4 are subsets of CD11b^+^ cells based on GFP expression with R1 and R3 being GFP^−^ cells, whereas R2 and R4 were infiltrating GFP^+^ cells. Both R1 and R2 were cells derived from the contralateral hemisphere, whereas R3 and R4 were derived from the ipsilateral hemisphere. To identify mononuclear phagocytes with the R1–R4 subsets, a refined analysis removed NK cells and neutrophils based on NK1.1 and Ly6G expression, respectively. The resulting CD11b^+^/CD45^+^/NK1.1^−^/Ly6G^−^ population was subdivided into R1’–R4’ subsets using the schema as the R1–R4 subsets. **d** Counts for CD11b^+^ cells in R1–R4. **e** Counts of CD11b^+^/CD45^+^/NK1.1^−^/Ly6G^−^ microglia and MDMs in R1’–R4’. Statistical significance was assessed with two-way ANOVA for isolation with Percoll gradient method (^**^ indicating *p* < 0.01), Student t-test for isolation with debris removal (^****^ indicating *p* < 0.0001), and with one-way ANOVA with post hoc Bonferroni comparison test for cell counts in **d** and **e** (with ^*^, ^**^, and ^****^, respectively, indicating *p* < 0.05, 0.01, and 0.0001)

To identify the peripheral origin of MDMs in the post-ischemic brain, we infused GFP^+^ splenocytes into adult mice subjected to middle cerebral artery occlusion (MCAO). Stroked mice underwent a splenectomy 3 days before killing and GFP^+^ splenocytes were adoptively transferred 1 day before killing (i.e., 2 days after spleen removal; Fig. 1a). The spleen is a reservoir of monocytes that are deployed after injury [30]. The absence of endogenous monocyte release for 48 h in asplenic mice facilitates the infiltration and visualization of the exogenously infused GFP^+^ splenocytes in the post-ischemic brain.

Flow cytometry analysis of the post-ischemic brain at 24 h after the splenocyte transfer showed a significant increase in total number of GFP^+^ cells within the ipsilateral hemisphere, relative to the contralateral hemisphere. This increase was observed at both 3 days (with infiltration during 2nd and 3rd day post-stroke) and 7 days (with infiltration during 6th and 7th day post-stroke; Fig. 1b). We initially isolated brain mononuclear phagocytes using a Percoll gradient according to a previously established method [1], but we found that incorporating a myelin debris removal step substantially increased the yield of GFP^+^ immune cells from post-ischemic brain tissue (Fig. 1b).

The 3-day survival group had a higher mortality rate than the 7-day group (34.8% and 20.8%, respectively). This increased mortality is likely due to an inability to meet the increased need for immune cell release from the spleen in asplenic mice during the first 3 days post-stroke. Because of this elevated mortality, all subsequent studies evaluating monocyte trafficking and function were performed at 7 days post-ischemia.

To analyze the CD11b^+^ population in the post-stroke brain, flow cytometry analyses divided cells into four subsets (R1−R4, Fig. 1c, Additional file 1: Table S1). R1–2 and R3–4 were, respectively, derived from hemispheres contra- and ipsi-lateral to injury. The R1 and R3 subsets consisted of CD11b^+^/GFP^−^ cells, whereas R2 and R4 were CD11b^+^/GFP^+^ cells (Fig. 1d). The R2 subset had a low number of cells, which reflects the lack of trafficking from the periphery into the non-injured, contralateral hemisphere. Moreover, the low cell numbers in R2 confirm that spleen removal did not promote non-specific infiltration of exogenous GFP^+^ cells. In the injured, ipsilateral hemisphere, stroke increased both R3 (CD11b^+^/GFP^−^) and R4 (CD11b^+^/GFP^+^) subsets (Fig. 1d). The R3 (GFP-) subset was a mix of resident cells and endogenous infiltrating cells during the entire post-stroke period, whereas R4 was composed of exogenously derived GFP^+^ cells that infiltrated into the brain during the 24 h post-transplantation period (Fig. 1d).

Because CD11b is also expressed by NK cells and neutrophils, we refined the flow cytometry gating to define microglia and MDMs as CD45^+^/CD11b^+^ cells that were also NK1.1^−^/LY6G^−^. The resulting CD11b^+^/CD45^+^/NK1.1^−^/LY6G^−^ population was subdivided as before and the subsets were renamed R1’−R4’ (Fig. 1e). This analysis found that monocytes were 58.6% of the R4’(GFP^+^) population, indicating that these are the predominant cell type infiltrating in the post-ischemic brain (Additional file 2: Table S2).

### MDMs display high expression of Ki-67 and CD11c and become a microglia-like phenotype in the ischemic brain

**Fig. 2:**
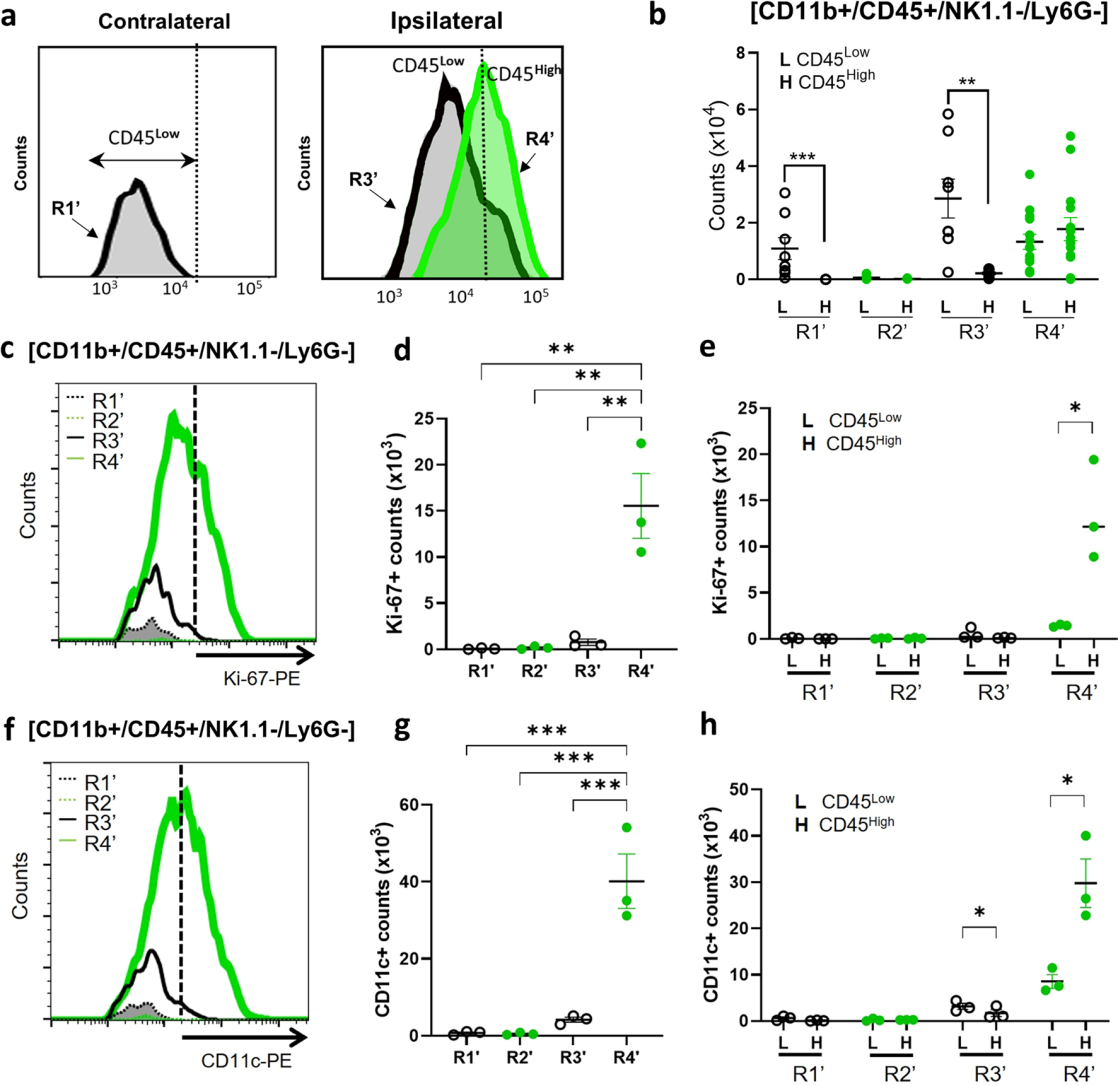
Analyses of CD45 subsets in MDMs and microglia at 7 days the post-ischemic brain. **a** Gating strategy for cell counts of CD11b^+^/CD45^+^/NK1.1^−^/Ly6G^−^ microglia and MDMs in R1’–R4’. **b** Cell counts for CD45^Low^ and CD45^High^ subsets within R1’–R4’. **c** Gating strategy for analysis of Ki-67 expression in R1’–R4’. **d** Cell counts of Ki67^+^/GFP^−^ cells in R1’ and R3’, as well as Ki67^+^/GFP^+^ cells in R2’ and R4’. **e** Counts of cells in **d** divided into CD45^Low^ and CD45^High^ expression. **f** Gating strategy for analysis of CD11c expression in R1’–R4’. **g** Cell counts of CD11c^+^/GFP^−^ cells in R1’ and R3’, as well as CD11c^+^/GFP^+^ cells in R2’ and R4’. **h** Counts of cells in **g** divided into CD45^Low^ and CD45^High^ expression. Statistical significance was assessed using one-way ANOVA with post hoc Bonferroni comparison test in **d** and **g**, whereas Student T-test was used in **b**, **e**, and **h** (with ^*^, ^**^, and ^***^, respectively, indicating *p* < 0.05, 0.01, and 0.001)

CD45 expression levels are typically used to distinguish CD45^Low^ microglia from CD45^High^ MDMs [31, 32]. The R1’ subset in the contralateral hemisphere was almost exclusively CD45^Low^, which represented the resident microglia in that hemisphere (Fig. 2a, b). In the R2’ subset, the absence of CD45^+^ cells indicated that there was no detectable trafficking from the periphery into the non-injured hemisphere. In the R3’ subset, like R1’, nearly all of the cells were CD45^Low^ cells, but unlike R1’, the cells in R3’ represented both resident microglia and endogenous (GFP^−^) CD45^High^ MDMs that converted to the CD45^Low^ phenotype. The R4’ subset contained exogenously derived (GFP^+^) MDMs that infiltrated into the ipsilateral hemisphere during the 6 days and 7 days post-stroke. Unlike the other subsets, R4’ had nearly equivalent numbers of CD45^High^ and CD45^Low^ MDMs (Fig. 2b), indicating that many of infiltrating monocytes converted to ‘microglia-like’ CD45^Low^ MDMs in the post-ischemic brain. These findings are consistent with prior reports of macrophage phenotypic plasticity in the CNS [15, 17].

Previous studies have suggested microglia can proliferate in the post-ischemic brain [33]. To investigate the proliferative capacity in MDMs, the R1’–R4’ subsets were probed for Ki-67 expression, a marker of proliferation (Fig. 2c). Ki-67^+^ cells were almost completely absent in R1’, R2’ and R3’ (Fig. 2d). The lack of proliferative capacity in endogenous MDMs within R3’ may be due to their early entry into the brain (0–2 days) prior to GFP + cell transfer and a change to a microglia-like phenotype. By contrast, R4’ had a substantial number of cells expressing Ki-67, and this expression was nearly exclusive to the CD45^High^ subset and not in CD45^Low^ subset (Fig. 2d, e), suggesting proliferation occurs in initially infiltrating MDMs. These findings indicate that MDMs, but not microglia, proliferate in the post-ischemic brain.

CD11c is a marker of disease-associated microglia [34], and a recent study identified a unique microglia subtype that expresses CD11c during the post-stroke secondary degeneration in the thalamus [35]. Our analyses in the post-ischemic brain found that CD11c^+^ cells were mostly in the CD45^High^ MDMs subset of R4’ (Fig. 2f-h). This observation suggests that CD11c^+^ cells in the post-stroke brain originate in the periphery and contribute to immunomodulatory functions in cerebral ischemia.

### MDMs are the major phagocytes in the post-ischemic brain

**Fig. 3:**
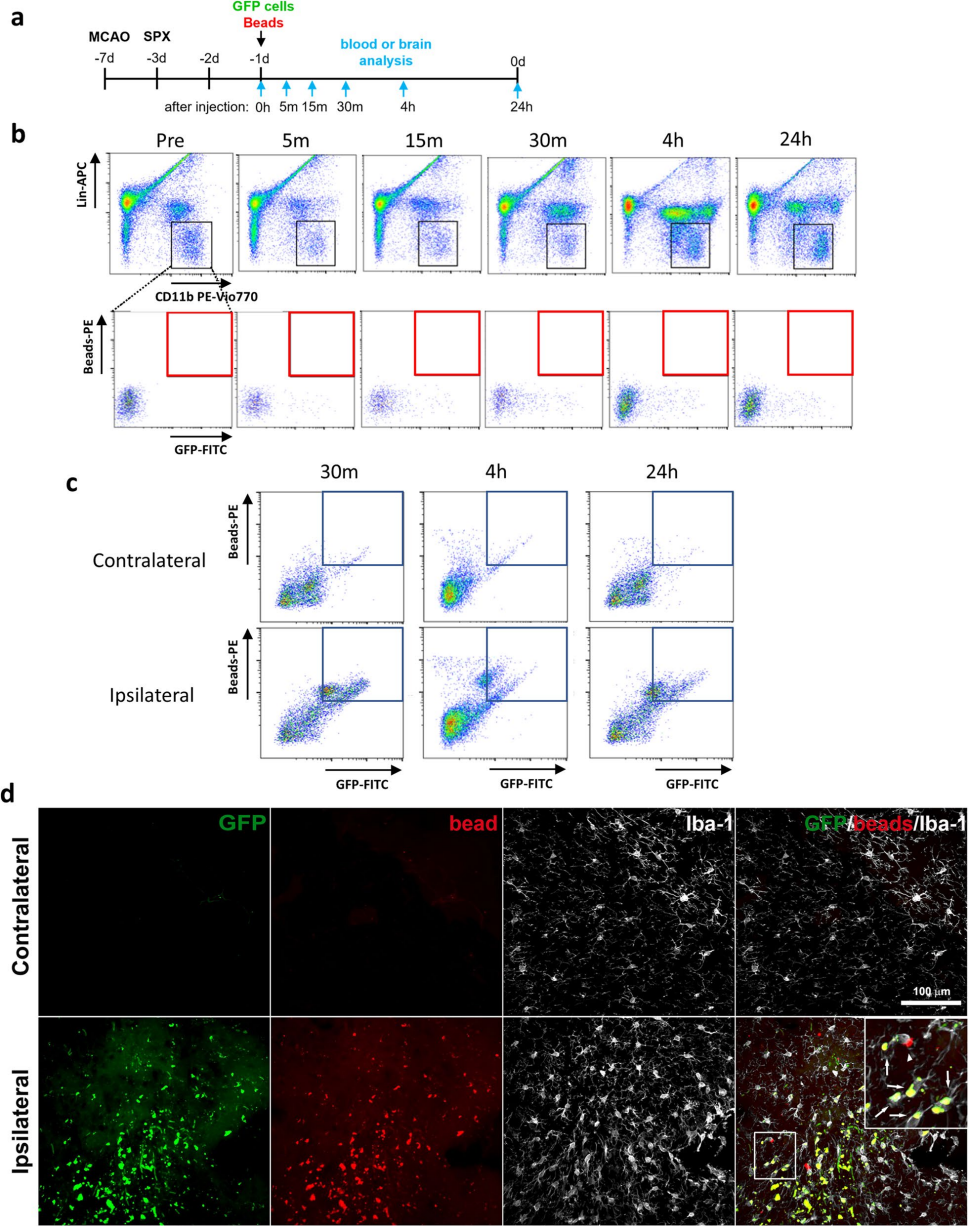
Assessment of phagocytosis in circulating monocytes and MDMs in the brain. **a** Experimental timeline. Asplenic mice were received infusion of GFP^+^ splenocytes and bead^580/605^ at 6 days after MCAO and 2 days after splenectomy (SPX). Following the infusion, blood and brain tissue were collected at several time points up to 24 h, as indicated by arrows. **b** Assessment of bead^+^/GFP^+^ population in circulating monocytes at baseline at 0 h (pre-infusion of GFP splenocytes and bead^580/605^; Pre) as well as at 5 m, 15 m, 30 m, 4 h, and 24 h post-infusion. Lin (lineage markers) is the mixture of antibodies against T cells (CD90.2), B cells (CD45R/B220), natural killer cells (NK1.1), and granulocytes (Ly6G). The monocyte population was identified by CD11b^+^/Lin^−^ cells (indicated by black boxes) and were further gated for GFP^+^/bead^+^] population (indicated by red boxes). **c** Assessment of MDMs [GFP^+^/bead^+^/CD11b^+^/CD45^+^] in the post-ischemic brain 30 m, 4 h, and 24 h following infusion of GFP splenocytes and bead^580/605^. MDMs are in the boxed region. Representative plots from three independent experiments per time point. **d** Immunofluorescence images of the striatum taken at 7 days post-stroke with arrows (white) indicating phagocytic MDMs [GFP^+^/bead^+^/Iba1^+^], arrowheads indicating either microglia or infiltrated endogenous non-tagged MDMs [GFP^−^/bead^+^/Iba1^+^], and * (aqua) indicating non-phagocytic MDMs [GFP^+^/bead^+^/Iba1^+^]. Scale bar = 100 µm

To evaluate the phagocytic activity of MDMs, we infused red fluorescence beads (beads^580/605^) into post-stroke mice receiving an adoptive transfer of GFP^+^ splenocytes (Fig. 3a). To establish whether GFP^+^ cells engulfed beads^580/605^ prior to entering to the brain, we examined whether circulating GFP^+^ cells contained beads^580/605^. This analysis did not find any GFP^+^/beads^580/605+^ cells among circulating monocytes [CD11b^+^/Lin^−^] during the 24 h period following the infusion of GFP^+^ cells and beads (Fig. 3b). By contrast, GFP^+^/beads^580/605+^ cells were abundant in ipsilateral hemisphere brain tissue during the 24-h period after infusion (Fig. 3c). Immunofluorescence analyses showed that the vast majority of Iba1^+^/beads^580/605+^ cells in the ipsilateral hemisphere were also GFP^+^, whereas GFP^+^ and/or beads^580/605+^ cells were not observed in the contralateral side (Fig. 3d). Although the most of MDMs were phagocytic, we found few non-phagocytic MDMs in the post-ischemic brain (Additional file 3: Fig S1). Together, these findings show that MDMs engage in phagocytosis upon entering to the ipsilateral hemisphere. engulf.

**Fig. 4.**
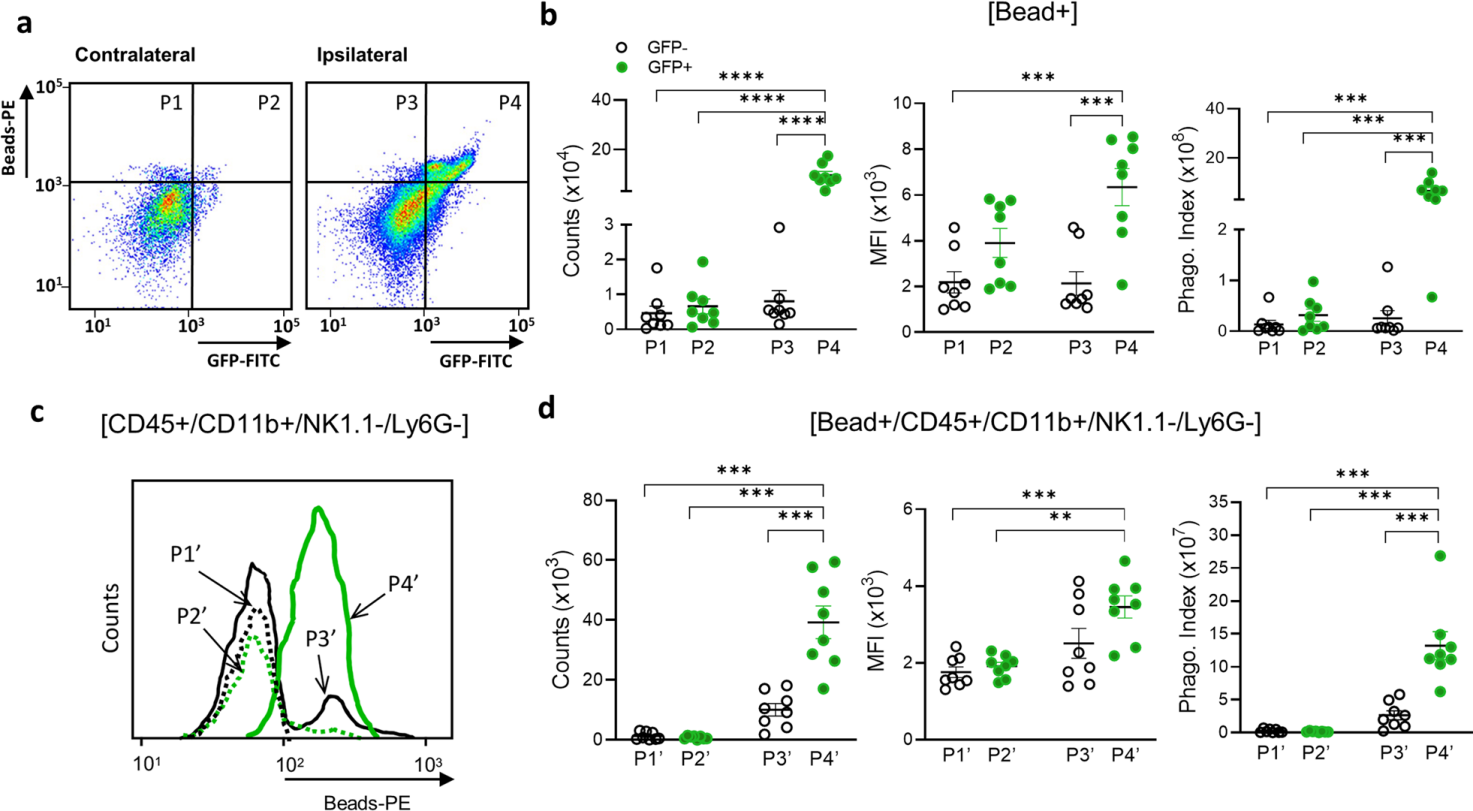
Ex vivo phagocytosis assays in MDMs and microglia in the post-ischemic brain. For phagocytosis assays, the cells were isolated in the post-ischemic brain of asplenic mice that received GFP + splenocytes and bead^580/605^ at 6th day after stroke. **a** Gating strategy for isolated cells containing GFP and /or bead^580/605^ with subsets P1–P4 containing bead^+^ phagocytes. **b** Measures of phagocytic activity in GFP^−^ (P1, P3) and GFP^+^ (P2, P4) cells within the contralateral (P1, P2) and ipsilateral (P3, P4) hemispheres. Phagocytic activity was monitored by counts of cells containing bead^580/605^ (counts), mean fluorescence intensity (MFI) of bead^+^ cells, and a phagocytic index (counts x MFI). **c** Cell count distribution of P1’–P4’ subsets, which contain CD45^+^/CD11b^+^/NK1.1^−^/Ly6G^−^ microglia and MDMs. **d** Measures of phagocytic activity for microglia (GFP^−^) and MDMs (GFP^+^) in the contralateral (P1’, P2’) and ipsilateral (P3’ P4’) hemispheres. Measures of statistical significance were determined by one-way ANOVA with post hoc Bonferroni comparison test (^**^, ^***^, and ^****^, respectively, indicating *p* < 0.01, 0.001, 0.0001)

To compare the relative contribution of phagocytic activity between MDMs and microglia, we used flow cytometry to measure the number of beads^580/605+^ cells (counts), the mean fluorescence intensity (MFI) of beads^580/605+^ cells, and the phagocytic index (counts x MFI) for beads^580/605+^ cells in the P1–P4 subsets (Fig. 4a). Bead + cells were absent in the contralateral hemisphere of bead-injected animals and as well as in both hemispheres from animals without bead injection. Similar to the previous flow cytometry analyses, P1 and P2 corresponded to the contralateral hemisphere, whereas P3 and P4 were in the ipsilateral hemisphere. In addition, P1 and P3 were GFP^−^ cells, whereas P2 and P4 were GFP^+^ cells. The number of phagocytic cells, MFI, and phagocytic index were low for the P1 and P2 subsets in the contralateral hemisphere (Fig. 4b). The P3 subset in the ipsilateral hemisphere also had few phagocytic cells as well as a low MFI and phagocytic index (Fig. 4b). By contrast, the P4 subset had a substantial number of phagocytic cells that also had the highest MFI and phagocytic index values (Fig. 4b). As done the previous flow analyses, we refined the analysis to distinguish between microglia and/or MDMs in the P1–P4 populations, and the respective CD11b^+^/CD45^+^/NK1.1^−^/LY6G^−^ subsets were relabeled as P1’–P4’ (Fig. 4c). This refined analysis found that exogenously derived MDMs (P4’) had the highest phagocytic activities compared to any other subset (Fig. 4d).

### MDMs display high phagocytic activity ex vivo

**Fig. 5:**
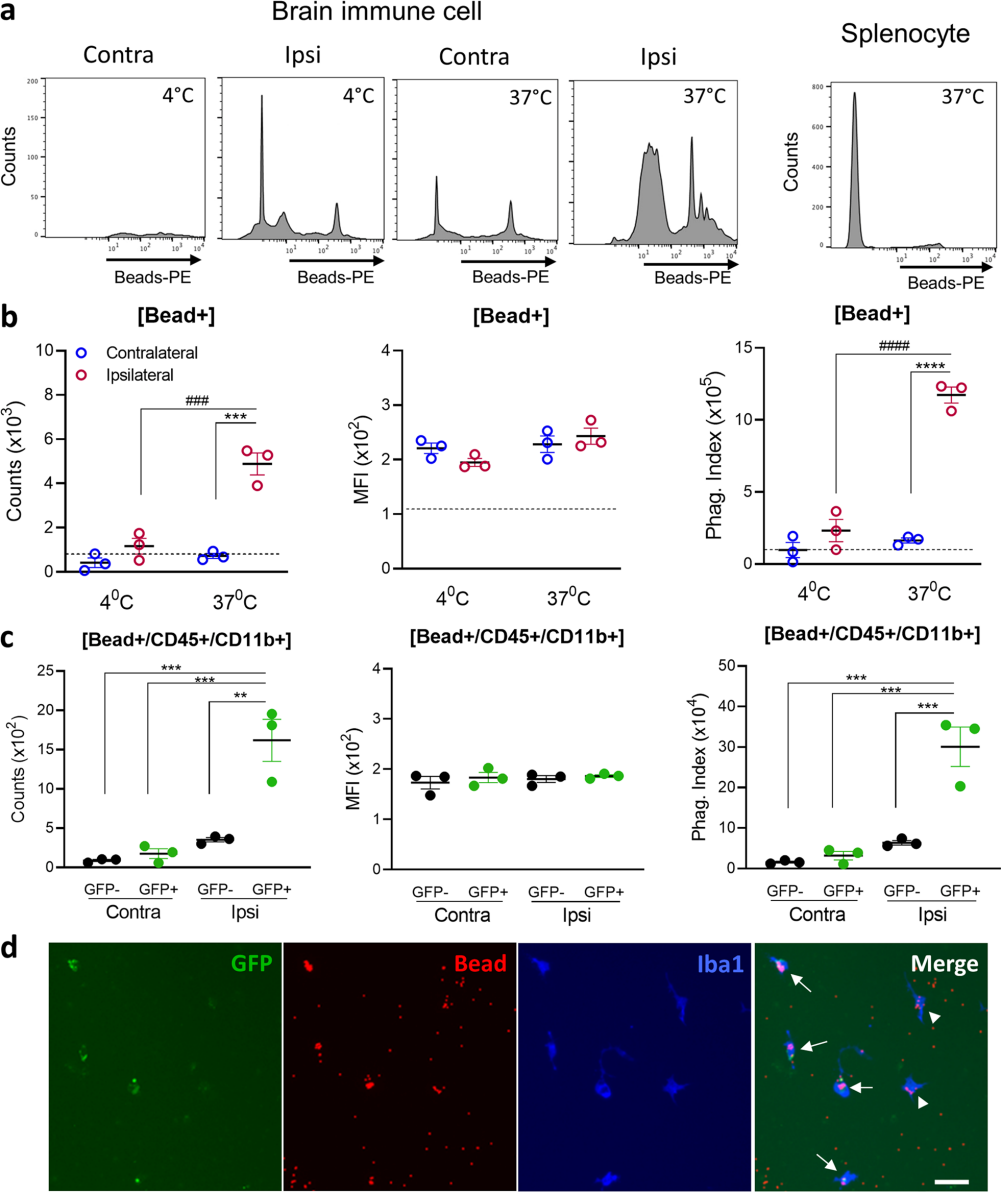
Ex vivo assessment of phagocytosis in MDMs and microglia. Ex vivo phagocytosis assays with cultured MDMs and microglia derived from the post-ischemic brain of asplenic mice that received GFP^+^ splenocytes and bead^580/605^ at 6th day after stroke. **a** Cell count distributions for GFP^+^ brain immune cells from contralateral (Contra) or ipsilateral (Ipsi) hemispheres cultured with bead^580/605^ at 4 °C or at 37 °C for 4 h. Cell counts with splenocytes cultured at 37 °C with bead^580/605^ were used as a negative control. **b** Measures of phagocytic activity for cultured brain immune cells derived from the contra- and ipsi-lateral hemispheres. Phagocytic activity was monitored by counts of cells containing bead^580/605^ (counts), mean fluorescence intensity (MFI) of bead^+^ cells, and a phagocytic index (counts x MFI). The dotted lines in the graphs indicate phagocytic activity of splenocytes incubated at 37 °C as a control. Statistical significance was determined using two-way ANOVA with a post hoc Bonferroni comparison test (^***^ and ^****^, respectively, indicate *p* < 0.001 and 0.0001 for the effect of stroke; ^###^ and ^####^, respectively, indicate *p* < 0.001 and 0.0001 for the effect of temperature). **c** Measures of phagocytic activity for CD11b^+^/CD45^+^ mononuclear phagocytes in brain immune cell cultures derived from the post-stroke contra- and ipsi-lateral hemispheres (*n* = 3/group). Statistical significance was determined using one-way ANOVA with a post hoc Bonferroni comparison test (^**^ and ^***^, respectively, indicate *p* < 0.01 and 0.001). **d** Representative immunofluorescence images of brain immune cells cultures in phagocytosis assays with arrows indicating phagocytic MDMs [GFP^+^/bead^+^/Iba1^+^] and arrowheads indicating phagocytic microglia or endogenous MDMs [GFP^−^/bead^+^/Iba1^+^]. Scale bar = 20 µm

To model MDM phagocytosis ex vivo, brain immune cells were isolated from mice 24 h after the infusion of GFP^+^ splenocytes and beads^580/605^ (Fig. 3a). Cells derived either from the contralateral hemisphere, incubated at 4 °C, or derived from splenocyte cultures had low numbers of beads^580/605+^ counts as well as minimal phagocytic index values (Fig. 5a, b). Cells from the ipsilateral hemisphere, however, had a significantly greater number of beads^580/605+^ cells without a corresponding change in the MFI, in addition to a substantially higher phagocytic index (Fig. 5a, b). Further analyses focusing on beads^580/605+^/CD45^+^/CD11b^+^ cells found the phagocytic activity of MDMs (GFP^+^) in the ipsilateral hemisphere was significantly higher than either resident microglia in the contralateral hemisphere or endogenous immune cells (GFP^−^) in the ipsilateral hemisphere (Fig. 5c). Immunofluorescence analysis for Iba-1 expression revealed that the most beads^580/605+^ cells were GFP^+^ MDMs (Fig. 5d). Together, these ex vivo analyses showed that MDMs are the major phagocytic cell type in the stroked hemisphere.

### Engulfment of beads in the post-ischemic brain induces MDM phenotype changes

**Fig. 6:**
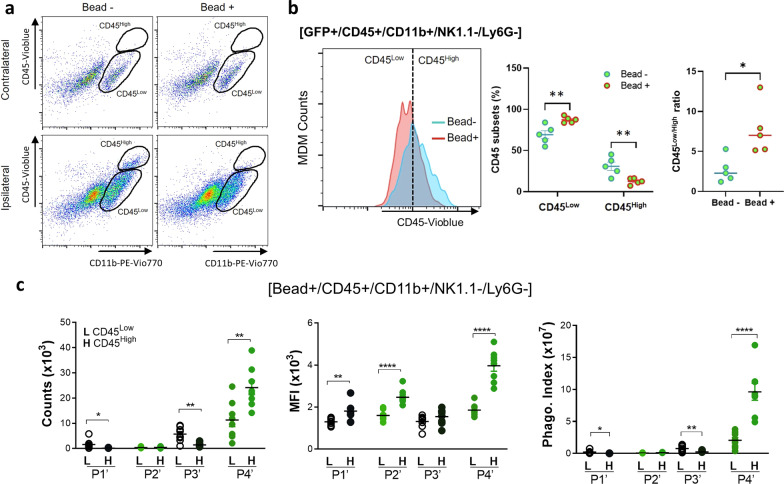
Assessment of MDM phenotype conversion upon phagocytosis. **a** Gating strategy for measuring phagocytic activity of CD45^+^/CD11b^+^/NK1.1^−^/Ly6G^−^ microglia and MDMs isolated from the contra- and ipsi-lateral hemispheres in asplenic, stroked mice 1 day following an infusion of GFP^+^ splenocytes that were incubated for 4 h with bead^580/605^. **b** Distribution of CD45^+^ subsets in GFP^+^ MDMs with or without bead^580/605^, counts of CD45^Low^ and CD45^High^ with or without bead^580/605^, and CD45^Low^/CD45^High^ ratio of MDMs in cultures either with or without bead^580/605^ (*n* = 5/group). Statistical significance was determined with Student T-test (^*^ and ^**^, respectively, indicate *p* < 0.05 and 0.01). **c** In vivo phagocytic activity of CD45^+^/CD11b^+^/NK1.1^−^/Ly6G^−^ microglia and MDMs in asplenic mice receiving infusions of GFP^+^ splenocytes and bead^580/605^ on the 6th day after stroke. Phagocytic activities were determined for CD45^Low^ (L) and CD45^High^ (H) cells (*n* = 8/group). Statistical significance was determined using Student T-test between CD45 subsets (^*^, ^**^, and ^****^, respectively, indicate *p* < 0.05, 0.01, and 0.0001)

CD45^High^ cells are generally accepted as MDMs in the post-ischemic brain [31, 32], but our in vivo analyses found that approximately 45% of MDMs in R4’ were CD45^Low^ (cell counts (× 10^3^) for CD45^High^ and CD45^Low^ were 22.33 ± 5.80 vs 17.86 ± 3.43, respectively; Fig. 2a, b). The unexpected high proportion of CD45^Low^ MDMs indicates that CD45^High^ MDMs change to a CD45^Low^ “microglia-like” phenotype in vivo. To test whether phagocytosis precedes and/or is required for this phenotypic conversion, brain immune cells were isolated from mice receiving an infusion of GFP^+^ splenocytes. Unlike the even distribution of CD45 subsets in vivo (Fig. 2b), 4 h cultures ex vivo resulted in a larger CD45^Low^ subset than CD45^High^ subset. Whether or not cells were incubated in the presence of beads^580/605^, CD45^High^ cells were selectively present only in cultures derived from the ipsilateral hemisphere (Fig. 6a). After a 4 h incubation with beads^580/605^, however, cells derived from the ipsilateral hemisphere had a significant increase in the number of CD45^Low^ cells, as well as corresponding reduction in the number of CD45^High^ cells (Fig. 6b). This reciprocal change in the distribution of CD45 subsets was also reflected by a shift towards a higher CD45^Low/High^ ratio in the cultures incubated with beads^580/605^ (Fig. 6b). These findings indicate that phagocytosis precedes, and is required, for the conversion of CD45^High^ MDMs to a CD45^Low^ microglia-like phenotype.

To confirm MDM phenotype conversion in vivo, phagocytic activities of CD45^+^/CD11b^+^/NK1.1^−^/Ly6G^−^ subsets (named P1’–P4’, as previously described and Fig. 4c) were measured in the post-ischemic brain of asplenic mice infused with GFP^+^ splenocytes and beads^580/605^. This analysis found relatively few phagocytic cells and a low phagocytic index for the contralateral P1’ and P2’ populations (Fig. 6c). The P3’ subset derived from the ipsilateral hemisphere, which contains mixture of microglia and endogenous GFP^−^ MDMs, had only a modest increase in the number of phagocytic cells within the CD45^Low^ population (Fig. 6c). This small number of phagocytic CD45^Low^ cells in P3’ likely includes endogenous CD45^High^MDMs that underwent phenotypic conversion in the ischemic brain. The P4’ subset, however, had significantly higher numbers of phagocytic CD45^High^ and CD45^Low^ MDMs (Fig. 6c). The MFI and phagocytic index were also significantly higher for the CD45^High^ MDMs in the P4’ subset (Fig. 6c). Despite the short time between the adoptive transfer of GFP^+^ splenocytes and analysis (only for 24 h), the substantial proportion of CD45^Low^ MDMs in the ipsilateral hemisphere suggests that CD45^High^ to CD45^Low^ phenotype conversion occurs relatively quickly (< 24 h). Moreover, the ex vivo and in vivo findings collectively demonstrate that MDMs, and not microglia, are the major phagocytic cell type in the post-ischemic brain, and that phagocytosis facilitates the conversion of MDMs to microglia-like phenotype in the post-ischemic brain.

## Discussion

Defining cell type-specific roles for microglia and MDMs in the post-stroke brain has been impeded by the overlapping expression of molecular markers, phenotypic heterogeneity within each cell type, and an ability of each cell type to modify their phenotype and function in response to CNS environments [15, 17, 36–39]. The adoptive transfer of GFP-tagged splenocytes used in this study allowed MDMs to be distinguished from resident microglia in the post-ischemic brain. This strategy also enabled us to define the origin and function of MDMs during acute phase of stroke. Stroke induces a massive infiltration of monocytes into the brain, and we showed that there is an equal distribution of CD45^High^ and CD45^Low^ subsets in the infiltrating MDMs. The unexpected and high proportion of CD45^Low^ MDMs indicates that CD45^High^ MDMs change to a CD45^Low^ microglia-like phenotype in the post-ischemic brain. Unlike microglia, MDMs in the post-stroke brain were proliferative, expressed CD11c, and were competent phagocytes. Additionally, CD45^High^ MDMs have higher phagocytic activity when compared to the CD45^Low^ subset. Moreover, the CD45^High^ to CD45^Low^ phenotype conversion in MDMs requires phagocytosis. Together, this study shows that MDMs are the major phagocytic cell type in the ischemic brain and have plastic phenotypes as part of tissue repair following cerebral ischemia.

Emerging evidence indicates that microglia and MDMs undergo phenotype changes and functional convergence in the CNS [15, 17, 39]. The conversion of CD45^High^ MDMs to a CD45^Low^ phenotype we observed in the post-stroke brain is consistent with these previous reports. Our studies, however, did not find compelling evidence for the converse conversion of microglia to an inflammatory MDM phenotype. The vast majority of GFP^−^ cells in the R3’ subset was CD45^Low^, but there was a small CD45^High^ sub-population (Fig. 2b). This small subset was likely endogenous MDMs that entered the injured hemisphere, but some cells could have possibly been endogenous microglia that acquired an MDM phenotype.

In an animal model of Alzheimer’s disease, CD45^High^ cells had a higher phagocytic capacity and expressed TREM2 and CD11c, both of which are markers that resemble disease-associated microglia [34, 40]. The origin of CD11c^+^ mononuclear phagocytes was not defined in this study, but a peripheral origin has been reported for a portion of CD11c^+^ dendritic cells in stroke [41]. In our analyses, we found that CD11c^+^ cells were predominantly CD45^High^ MDMs (R4’; Fig. 2f−h). Thus, our findings indicate that microglia do not acquire a disease-modifying MDM phenotype in the post-stroke brain. Since CD11c^+^ dendritic cells play a role in inducing Th1 cell-mediated immunity and exert protective effects [41, 42], we speculate that MDM-derived CD11c^+^ cells modulate adaptive immune function following stroke. Previous studies have also reported that microglia proliferate in ischemic brains [43, 44], but we found that Ki-67^+^ cells were abundant in the CD45^High^ MDMs of R4’ and only minimal in the R1’–R3’ subsets (Fig. 2c-e). This observation indicates that proliferation is restricted to infiltrating MDMs.

Clearing cellular debris by phagocytes in the injured CNS is critical for tissue repair and remodeling [1, 45], but establishing the relative phagocytic activity of MDMs and microglia has been challenging. In our study, we found that MDMs (P4’) were the major phagocytes in the ischemic brain (Fig. 4d), with a higher phagocytic capacity in CD45^High^ MDMs compared to CD45^Low^ MDMs (Fig. 6c). This finding is consistent with a report that microglia-induced phagocytosis can be suppressed by peripherally derived macrophages [20]. Differences in phagocytic capability between CD45^High^ and CD45^Low^ subsets within the injured brain, in conjunction with phenotype changes, posed an intriguing question as to whether phagocytosis is necessary for the phenotype conversion in MDMs. Our findings support the hypothesis that phagocytosis has a causal role in the CD45^High^ and CD45^Low^ conversion within MDMs.

A limitation of this study is that the adoptive transfer procedure to infuse GFP-tagged splenocyte to asplenic mice does not truly reflect post-stroke in normal status. The rationale for removing the spleen 2 days prior to cell transfusion was to allow time for the reduction of the endogenous monocyte pool in the blood, which facilitates trafficking of the exogenously infused GFP-tagged splenocytes. Parabiosis can provide an in vivo tool to study MM trafficking. Sharing hematopoietic cells between parabionts (e.g., WT and GFP mice), however, will greatly diminish the number of fluorescently tagged MMs in circulation. This reduction will likely result in high endogenous non-tagged MM trafficking into the brain, when compared to our model system with GFP + splenocyte transfer into asplenic mice. An additional caveat in our approaches is the underestimation of the number of MDMs that infiltrate and/or accumulate during the post-stroke survival periods due to limiting the post-infusion time to only 24 h. While bone marrow transplantation between Wt and GFP-tg mice provides the number of MDMs infiltrated over time, the approach would not provide in situ trafficking at a given post-stroke time point. Thus, the advantage of in situ trafficking used in this study is the ability to clearly define the origin and function of temporally trafficking MDMs. In summary, the ability to distinguish MDMs from microglia, the study revealed that MDMs are the major phagocytes, not microglia, in the ischemic milieu and that they undergo phenotype changes to microglia-like cells upon phagocytosis. The study provides a defined expansive role of MDMs in phagocytosis and tissue repair in cerebral ischemia.

## Supplementary Information

**Additional file 1: Table S1. **

**Additional file 2: Tables S2. **

**Additional file 3: Fig S1.**

## Data Availability

The data generated during and/or analyzed during the current study are available from the corresponding author on reasonable request.

## Abbreviations

CCR2: C–C motif chemokine receptor type 2
CX3CR1: C–X3–C motif chemokine receptor type 1
GFP: Green fluorescence protein
MCAO: Middle cerebral artery occlusion
MDMs: Monocyte-derived macrophages
NK: Natural killer
