# Supplementary material for: Phagocytosis converts infiltrated monocytes to microglia-like phenotype in experimental brain ischemia
Source: J Neuroinflammation. 2022 Jul 18;19:190. doi: 10.1186/s12974-022-02552-5 (PMC9295522; doi:10.1186/s12974-022-02552-5)
Supplement: Supplementary file 1 — Additional file 1: Table S1. [file 12974_2022_2552_MOESM1_ESM.pdf]

Supplementary Table 1. GFP+ cell numbers in brain at 7d post-ischemia.

Cell type	Cell Numbers (x 10^3)	
	Contralateral	Ipsilateral
Total splenocyte [GFP+]	4.19 ± 0.79	83.13 ± 13.53
Lymphocyte [GFP+/CD11b-]	1.73 ± 0.66	12.16 ± 3.87
MDM, NK cell, Neutrophil [GFP+/CD11b+]	0.93 ± 0.32	54.85 ± 7.29
MDM [GFP+/CD11b+/CD45+/NK1.1-/LY6G-]	0.89 ± 0.31	32.17 ± 6.29
NK cell, Neutrophil [GFP+/CD11b+/CD45+/NK1.1+/LY6G+]	0.02 ± 0.01	10.79 ± 2.66
