# Supplementary material for: Phagocytosis converts infiltrated monocytes to microglia-like phenotype in experimental brain ischemia
Source: J Neuroinflammation. 2022 Jul 18;19:190. doi: 10.1186/s12974-022-02552-5 (PMC9295522; doi:10.1186/s12974-022-02552-5)
Supplement: Supplementary file 2 — Additional file 2: Tables S2. [file 12974_2022_2552_MOESM2_ESM.pdf]

Supplementary Table 2. Composition of immune cells in R3 and R4 region at 7d post-ischemia.

	Cell Numbers (x 10^3)	(%) in R3 or R4
R3<br>[GFP-/CD11b+]	43.22 ± 8.68	
R3': MDM, microglia<br>[GFP-/CD11b+/CD45+/NK1.1-/LY6G-]	30.92 ± 7.13	71.5
NK cells, Neutrophils<br>[GFP-/CD11b+/CD45+/NK1.1+/LY6G+]	3.13 ± 0.85	7.2
R4<br>[GFP+/CD11b+]	54.85 ± 7.29	
R4': MDM<br>[GFP+/CD11b+/CD45+/NK1.1-/LY6G-]	32.17 ± 6.29	58.6
NK cells, Neutrophils<br>[GFP+/CD11b+/CD45+/NK1.1+/LY6G+]	10.79 ± 2.66	19.7
