# Supplementary figures and images for: Phagocytosis converts infiltrated monocytes to microglia-like phenotype in experimental brain ischemia
Source: J Neuroinflammation. 2022 Jul 18;19:190. doi: 10.1186/s12974-022-02552-5 (PMC9295522; doi:10.1186/s12974-022-02552-5)

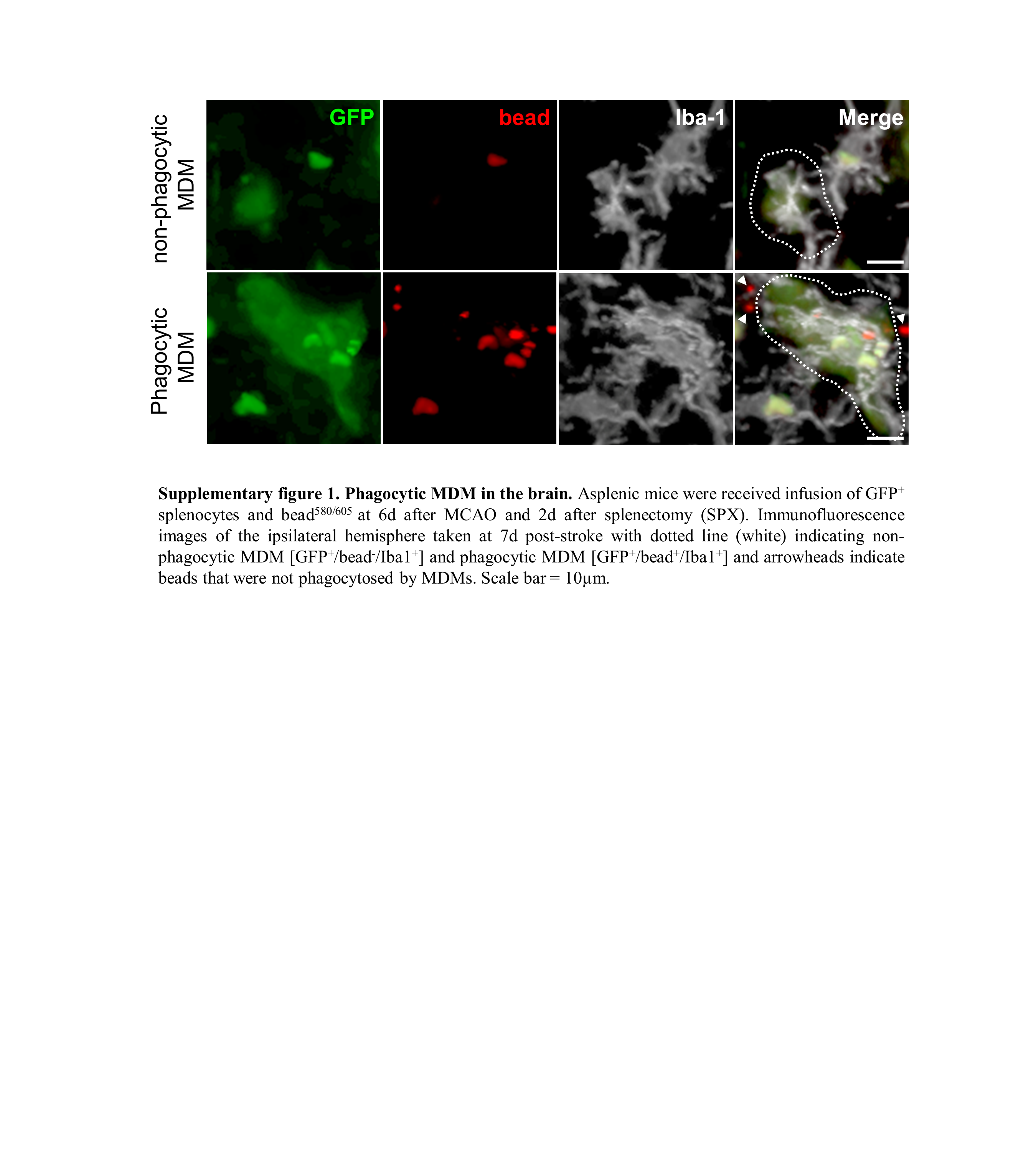

Supplement: Supplementary file 3 — Additional file 3: Fig S1. [file 12974_2022_2552_MOESM3_ESM.tif]
